# Avian extinctions induced by the oldest Amazonian hydropower mega dam: evidence from museum collections and sighting data spanning 172 years

**DOI:** 10.7717/peerj.11979

**Published:** 2021-08-18

**Authors:** Luiza Magalli Pinto Henriques, Sidnei Dantas, Lucyana Barros Santos, Anderson S. Bueno, Carlos A. Peres

**Affiliations:** 1 Coordenação de Pesquisas, Instituto Nacional de Pesquisas da Amazônia, Manaus, AM, Brazil; 2 Coordenação de Zoologia, Museu Paraense Emílio Goeldi, Belém, PA, Brazil; 3 Coordenação Espacial da Amazônia, Instituto Nacional de Pesquisas Espaciais, Belém, PA, Brazil; 4 Instituto Federal de Educação, Ciência e Tecnologia Farroupilha, Júlio de Castilhos, RS, Brazil; 5 School of Environmental Sciences, University of East Anglia, Norwich, Norfolk, United Kingdom

**Keywords:** Avifauna, Forest fragmentation, Long term impacts of dams, Threatened species, Tocantins River, Citizen science

## Abstract

Hydroelectric dams represent an emergent threat to lowland tropical forest biodiversity. Despite the large number of operational, under-construction, and planned hydroelectric dams, their long-term effects on biodiversity loss are still poorly documented. Here, we investigate avian extinctions resulting from the Tucuruí Hydroelectric Reservoir (THR), the oldest Amazonian mega dam, which impounded the Tocantins River in 1984. Our avian inventory—based on several sampling methods (mist-netting, point-counts, boat census and qualitative surveys) during 280 days of fieldwork from 2005 to 2007—was combined with an exhaustive search of museum vouchers and digital online databases of citizen science from the lower Tocantins River to identify long-term trends in species persistence and extinction in the THR influence area. The regional avifauna was comprised of 479 species, 404 of which were recorded during our fieldwork. Based on recent and historical records spanning 172 years, we found evidence for likely extinctions at THR influence area for 53 (11.06%) species that have remained entirely unreported since 1984. We were further able to estimate extinction probabilities for 20 species; 15 species were considered to be extinct, including *Psophia interjecta* and *Pyrilia vulturina* that are red-listed by IUCN. Our study serves as a baseline for avifaunal monitoring in the THR influence area and shows that degree of habitat specialization is a key factor in determining species extinctions caused by nonrandom habitat loss from either inundation or deforestation. Avian species extinctions will most likely continue across the area affected by the reservoir as a direct impact of alluvial forest loss and ongoing habitat degradation of upland forests.

## Introduction

The hydropower sector is one of the key drivers of human-induced biodiversity loss in Amazonia, damming rivers, transforming landscapes and riverscapes through changes in water flow and creating several large hydroelectric reservoirs (Lees et al., 2016). These reservoirs destabilize the aquatic environment by changing the hydrological regime from lotic to lentic with several short to long term landscape-scale cumulative synergistic effects, including reduced water quality, loss of the natural inundation pulse, reduced sediment transport, microclimatic changes and modifications in the biotic composition that impact both aquatic and terrestrial biodiversity (Pringle, Freeman & Freeman, 2000; Finer & Jenkins, 2012; Lees et al., 2016).

For the Amazonian avifauna, the main environmental impact is the permanent flooding of riparian habitats (alluvial forest, river beaches and sandbars, river islands, and rocky outcrops), which eliminates reproductive, feeding, nesting and roosting sites for many bird species (Lees et al., 2016). The structural complexity and productivity of alluvial vegetation of these habitats define local avian diversity and species composition, and it has been estimated that at 15% of the Amazonian non-aquatic avifauna is restricted to habitats created by rivers (Remsen & Parker, 1983). Furthermore, prior to the onset of river damming, the construction of the associated infrastructure (including access roads, transmission lines, and urban settlements) accelerates the process of human occupation with habitat degradation and forest fragmentation, which also have lasting impacts on avian population persistence (Ferraz et al., 2003; Stouffer, Strong & Naka, 2009; Stouffer et al., 2011).

Although hydroelectric dams have major impacts on bird populations, precious few studies have investigated the long-term impacts of major hydroelectric dams on the long-term persistence of tropical forest bird population (Wu et al., 2019). In Amazonia, previous studies examined either short-term changes (up to 7 years) in species richness and patterns of local abundance or long-term changes (22–29 years) in avian assemblages. These studies show a reduction in overall diversity and an increase in generalist species (*e.g.*, Terborgh, Lopez & Tello, 1997; Cosson et al., 1999; Claessens, 2002; Feeley, 2003; Feeley et al., 2007; Aurélio-Silva et al., 2016; Bueno et al., 2018; Bueno & Peres, 2019; Bueno & Peres, 2020). However, most of these studies were based on post-damming information obtained long after the reservoir was filled. Analysis that combines baseline data on species occurrence prior to dam construction, and detailed, continuous, and long-term data quantifying species extinctions caused by major infrastructure projects are still lacking. These analyses are required to determine the extent of avian extinctions because extinction lag times can vary from species to species resulting in “extinction debts”, with low-viability populations often requiring decades to become extirpated (Tilman et al., 1994). There is an urgent need to fill this knowledge gap because of the ambitious hydroelectric development plans for Amazonia that include hundreds of additional dams (Lees et al., 2016; Latrubesse et al., 2017).

The Tucuruí Hydroelectric Dam, which impounded the Tocantins River in 1984, is the oldest mega dam of Amazonia. Its planning and construction took place during the military dictatorship that ruled Brazil between 1964 and 1985. This authoritarian institutional context and the absence of environmental policies for the hydroelectric sector resulted in deficient and severely criticized environmental impact studies (Barrow, 1988; Fearnside, 2001), within which a comprehensive avifaunal study was no exception. Located in the eastern of the ‘Arc of Deforestation’ and encompassing both the Xingu and Belém Lowland Endemism Areas (Silva, Rylands & Da Fonseca, 2005), this region is included as a priority area for conservation in the Amazon basin based on the distribution of threatened bird species (Bird et al., 2011).

To work around data deficiencies and make long-term approaches feasible, several studies have empirically assessed predictions of avian assemblage changes in response to forest loss using both recent and historical records compiled from museum specimens and the literature across the Neotropical realm (see Kattan, Alvarez-Lopez & Giraldo, 1994; Christiansen & Pitter, 1997; Robinson, 1999; Ribon, Simon & Mattos, 2003; Moura et al., 2014; Cavarzere et al., 2017).

Here, we quantify the patterns of avian extinctions at the Tucuruí Hydroelectric Reservoir influence area. Our study was based on museum specimens collected during historical ornithological expeditions to the Tocantins River valley, which began in 1848, systematic avian surveys we conducted in this region over three years (2005–2007) and search of recent records from online citizen science databases. We present an updated species checklist, including detailed documentation of digital vouchers deposited online and all museum collections of the avifauna of the lower Tocantins River valley. More specifically, we asked the following questions: (1) How many and which species in the Tucuruí Hydroelectric Reservoir influence area detected before 1984 were no longer detected during the last 36 years? (2) What is the confidence level of extinction events of these undetected species? (3) Which species are most vulnerable to extinction? (4) Can some species in the novel Tucuruí Hydroelectric Reservoir avifauna be considered as either invasive or colonizers?

## Materials & Methods

### Study area

**Figure 1:**
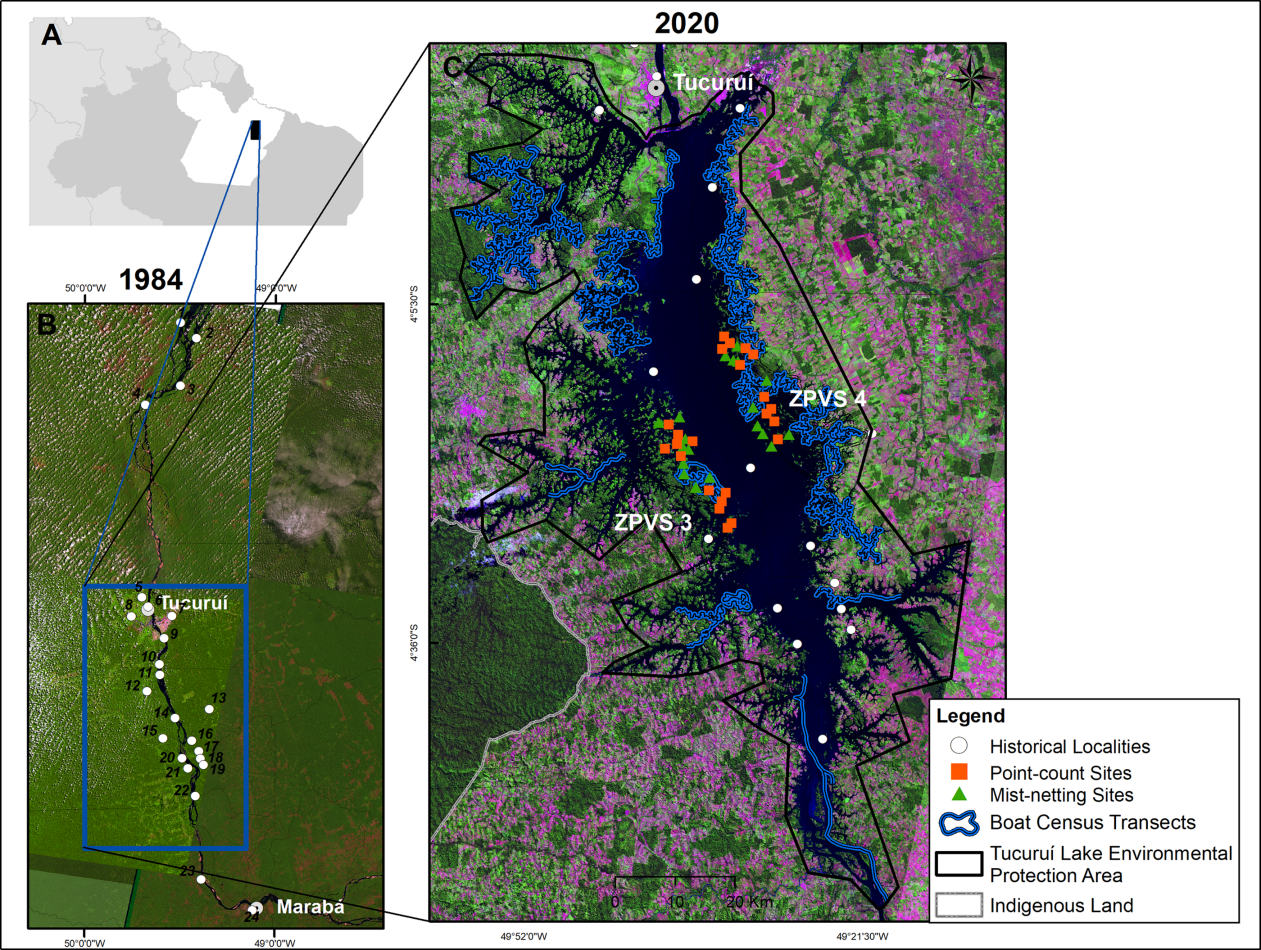
Map of the study area in eastern Brazilian Amazonia. Eastern Brazilian Amazonia (A). Satellite images showing the lower Tocantins River prior to the completion of the Tucuruí Dam in 1984, with historical ornithological localities (B). The actual shape with the Tucuruí Hydroelectric Reservoir within the Tucuruí Lake Environmental Protection Area (C), indicating the two Wildlife Conservation Zones (ZPVS 3 and 4). The larger forest patch is the Parakanã Indigenous Land. Image credit: U. S. Geological Survey at https://earthexplorer.usgs.gov/.

This study was conducted in the Tucuruí Hydroelectric Reservoir (THR) influence area in the State of Pará, northern Brazil (3°43′ to 5°15′S; 49°12′ to 50°00′W). The Tucuruí Dam, which impounded the eastern Amazonian Tocantins River in 1984, included a 4,000 MW installation capacity power station in the first phase, 72-m above sea level (Tucuruí-I) (Fearnside, 2001). In the second phase from 2002, the installed capacity increased to 8,370 MW, 74-m above sea level (Tucuruí-II). Raising the water level from 72 to 74 m increased the inundated forest area from 2,247 km^2^ in 1984 to ∼2,918 km^2^ in 2002 (Eletronorte, 1989; Fearnside, 2001). This resulted in the creation of some 2,200 forest islands on higher elevation terrain (Fig. 1).

The regional climate is markedly seasonal, with a rainy season from December to May and a dry season from June to November (Alvares et al., 2013). Mean annual precipitation and temperature are 2,354 mm and 27.5 °C, respectively (Alvares et al., 2013). The vegetation is typical of Amazonian *terra firme* forests, containing 80–90% forest cover and an understory dominated by several palm species (Ferreira et al., 2012).

In 2002, the Tucuruí Lake Environmental Protection Area (*APA Lago de Tucuruí*, in Portuguese) was implemented spanning an area of 568,667 ha. According to Brazilian Protected Areas legislation (SNUC, 2000), an Environmental Protection Area (APA) is a sustainable-use reserve (IUCN category V) designated as a mosaic to meet both the interests of local communities and wildlife conservation. Around 72% of the forested area has been lost from 1984 to 2017 within this APA, mostly due to forest conversion into cattle pastures (Velastegui-Montoya, De Lima & Adami, 2020).

When the area was flooded in 1984, thousands of terrestrial and arboreal vertebrates were rescued and released into two specific areas of remaining, named Zones of Wildlife Protection (ZPVS 3 and ZPVS 4), where fishing, hunting and human settlements were banned, as enforced by environmental agents from ELETRONORTE, the ELETROBRAS subsidiary company for public power services in the northern Brazil. ZPVS 3, on the left bank, is an area of 37,672 ha where 28.5% is flooded, 60% of which has native vegetation. ZPVS 4, on the right bank, spans an area of 22,731 ha, 59% of which is flooded and 39% is native vegetation remnants (Fig. 1).

We carried out bird inventories along most of the reservoir margins. The forest avifauna was intensively sampled in forest areas located on 39 islands ranging in size from 3.4 to 2,551 ha (mean ± SD = 169.3 ±488.8 ha) in both ZPVS 3 and ZPVS 4 using mist-netting and point count (Fig. 1). Waterbirds, birds associated with riparian habitats, and conspicuous species such as parrots, eagles and falcons were sampled in most of the reservoir area by boat censuses (Fig. 1).

### Avian species inventory

#### Primary data

A variety of methods was used to observe, identify, and collect birds within the THR influence area to produce a species checklist from modern surveys (SECTAM provided full approval for this research under license number 013/2005):

(a) *Mist-netting*. Mist-nets (12 m × 2.8 m, 36-mm mesh) were used to sample eight islands and two sites within a large pseudo-control island (1,823 ha) at ZPVS 3, and eight islands and two sites within a large pseudo-control island (2,551 ha) at ZPVS 4. We used four net-lines with eight nets (*i.e.,* 32 nets) on islands larger than 30 ha and sites within pseudo-control islands, and two net-lines with eight nets (*i.e.,* 16 nets) on islands smaller than 20 ha. Mist-net lines were operated simultaneously for two consecutive days of a netting session from 06:00 h to 14:00 h on the first day and 06:00 h to 12:00 h on the second day. Data were collected during three field campaigns in 2005 on both riverbanks: 10–26 July, 23 September to 4 October and 9–20 November in ZPVS 4, on the right bank; and 14–29 August, 10–21 October and 9–21 December in ZPVS 3, on the left bank.

(b) *Point counts*. Point count surveys were conducted where mist-netting took place, and at 23 additional sites for a total of 20 sites in ZPVS 4 and 20 sites in ZPVS 3. To maximize spacing between samples, point counts conducted throughout islands or along 2.5-km transects on the two largest islands (>1,800 ha), which were defined as pseudo-control island sites, to maximize spacing between samples. The number of point count stations per island ranged from 2 to 33 (Table S3). Timed point counts were carried out between 06:30 h and 10:00 h along each transect. Two observers spent 10 min at equidistant points along each transect, each of which separated by 200 m. All bird detections were recorded during point counts (using a Sony TCM 5000 recorder and a semi-directional microphone) and unknown vocalizations were subsequently checked against known calls. All uncertain identifications were removed from the analysis. Data were collected during six field campaigns: 6–25 August and 12–29 November in 2006; and 4–22 March, 12 April–1 May, 14–31 July and 22 September–10 October in 2007.

(c) *Boat census*. Surveys were carried out in a 40-HP outboard travelling at a velocity of 10 km/h, with each census transect recorded and mapped with a GPS. Boat census were conducted on a daily basis, from 06:30 h to 11:00 h. Birds were recorded with 10 ×40 binoculars and annotated on field worksheets, with each species checklist resulting from 30 min of observation, when a new list was initiated. For every sighting, the species and number of individuals were recorded. Each survey was conducted over a short period but covered a very large area. Data were collected during 11 field campaigns: 7–13 April, 06–11 August, and 19–28 November in 2005; 5–10 May, 7–12 July, and 17–24 November in 2006; and 8–17 June, 22–31 July, 25 August–03 September, 16–25 September, and 11–20 November in 2007.

(d) *Qualitative surveys.* We conducted qualitative surveys along transects used for quantitative surveys or during commuting travel by speedboat between sampling sites. During these surveys, one or two observers recorded all bird individuals and species sighted or heard. Most of these observations were conducted from 30 min before sunrise to 11:30 h, which represents the daily peak activity of birds and ensured the detection of both nocturnal and diurnal species. On many days, beginning at 15:00 h, we also sampled areas near the base camps, including intensive searches for nocturnal species until 22:00 h.

Sampling sufficiency per sampling method (mist-netting, point-counts, and boat censuses) was represented by individual-based rarefaction curves produced with 1,000 bootstrap replicates generated in the *iNEXT* R package (Hsieh, Ma & Chao, 2016; R Core Team, 2016). Sampling completeness per riverbank was quantified as a percentage between the observed and the estimated total number of species based on the Chao1 estimator (Chao, 1984).

#### Secondary data

The main source of specimen data came from digitized records of study skins deposited at Museu Paraense Emilio Goeldi (MPEG), the oldest Natural History Museum in Amazonia. This collection contains voucher specimens of species previously collected in the region since the early 20th century, including a large systematic collection conducted in 1978 and 1984, during the pre-flooding phase of the reservoir. Other five North American institutions with data from the Tocantins River area were also consulted *via* Vertnet (http://vertnet.org) to search for historically-collected specimens and retrieved records: American Museum of Natural History (AMNH), Harvard Museum of Comparative Zoology (MCZ), Field Museum of Natural History (FMNH), Carnegie Museum of Natural History (CM) and Los Angeles County Museum (LACM). Collecting localities were mapped using the coordinates provide by Paynter & Traylor (1991). Species for which we could identify a voucher specimen from the lower Tocantins River but could not be identified as a voucher from the THR influence area were moved to a hypothetical checklist of THR (Table S1).

Recordings on the Brazilian avian photo archive WikiAves, global avian sound library xeno-canto, and e-Bird were searched for all municipal counties around the THR area (Breu Branco, Goianésia do Pará, Jacundá and Nova Ipixuna, on the right bank, and Tucuruí, Novo Repartimento and Itupiranga, on the left bank); the catalogue number of photos, songs and complete species checklists (*i.e.,* field visits in which all species detected and positively identified by an observer were recorded) and observer identity are provided in Appendix S1). Species for which we identified vouchers for these counties but could not be identified as vouchers from the THR influence area were also moved to the hypothetical THR checklist (Table S1). Possible new records for the THR avifauna from e-Bird lists without permanent evidence (*i.e.,* photographic or tape-recording) were also included in the hypothetical THR checklist. The same procedure was adopted for species expected only for the right bank of Tocantins River and recorded for the left bank. We assessed the three online databases several times throughout the development of this study (last accessed on 21 April 2021).

#### Species checklist

The species list follows the classification and nomenclature adopted by Piacentini et al. (2015). Those species with seasonally variable abundance were designated as “migrant” and include both boreal (present during October–April) and austral migrants (present during April–September). Some species designated as “unexpected” may be either low-density, sporadic residents, vagrants or invasive (Appendix S1). Several ecological traits were determined for each species based on the authors’ primary observations or based on variables sources (*e.g.,* Stotz et al., 1996; Henriques, Wunderle & Willig, 2003; Wilman et al., 2014). We also compiled a checklist of avian taxa classified as currently or historically threatened and occurring in the THR influence area using the IUCN Red List (IUCN, 2019) and the Brazil threatened species list (ICMBio, 2018).

#### Extinction hypotheses and extinction risk

We used a presence-absence analysis considering 1984 as the cut-off pre-flooding year to infer local extinctions. Species unrecorded since 1984 were candidates for local extinctions, given the rigorous and intensive inventories we conducted over three years (2005–2007) combined with exhaustive search of consistent records in online citizen science databases (WikiAves, xeno-canto and e-Bird) and the severe environmental impacts caused by the THR. Species presence records were assumed to be reliable, but the same could not be said for absence records; indeed, failing to detect a species does not ensure that the species is entirely absent from our study area. To infer extinction, we follow the same procedure of Moura et al. (2014) for Eastern Amazonia that used the equations of Solow (1993) and Solow (2005): *p* = (*t*_*n*_/*T*)^*n*−^^1^, where *T* is the difference between the first sighting and the target year 2020 (the endpoint of our search in online citizen science databases); and *T*_*E*_ = (*n* + 1/*n*)*t*_*n*_, where *T*_*E*_ is the expected year of extinction and *t*_1_ is the first record (for species yielding at least 5 independent records). The confidence interval for *T*_*E*_ was calculated as ${T}_{E}^{U}$ = t _*n*_/*α*^1^^/*n*^, where *α* = 0.05.

Complementarily, we use the Preference Ranking Organization Method for Enrichment Evaluation (PROMETHEE) to provide a rank of extinction risk for the entire THR avifauna. PROMETHEE is a multi-criteria decision analysis (MCDM) that quantifies the degree of preference of one object compared with another for each variable (Brans & Vincke, 1985). An overall ranking is then constructed using the data matrices for each variable.

Vulnerability to extinction may depend on the level and type of threat and interactions among multiple factors (*e.g.*, biogeographic, morphological, and behavioral) (Pearson et al., 2010; Sodhi, Liow & Bazzaz, 2004). Considering that the interactive model including habitat breadth, geographic range size and abundance was the best supported in our island occupancy modeling analysis of the THR avifauna (Bueno et al., 2018), we combine these three variables or dimensions of rarity (*sensu* Rabinowitz, 1981) with a fourth, the relationship between the expected and observed occurrence. The first two variables were extracted from IUCN (2019) and the two last variables were extracted from our fieldwork and historical records. Because we used several census methods, we use a quantitative metric of rarity (*e.g.*, Camargo, 1993) to categorize species as either “uncommon” or “common” (*i.e.,* not rare) to dichotomize species abundance (Henriques, Wunderle & Willig, 2003). Based on species richness, a species is considered as rare if its relative abundance is less than the average relative abundance of all species in a community or assemblage (*i.e.,* <1/S, where S = richness). Species recorded only during qualitative surveys were considered as “uncommon” (Table S2). We attributed the same weight for all variables. In addition, we present the local incidence matrix of the most vulnerable species of forest birds found on 39 islands in the THR influence area, in which the islands are ordered from largest to smallest and species are ordered from most to least abundant. Our intent was to assess species vulnerability to forest fragmentation in terms of a species local extinction risk across the entire set of forest patches remaining across the landscape.

## Results

### Avian species inventory

Our assessment in the THR influence area resulted in 479 species representing 74 families Appendix S1. Of this total, 355 species already had at least one voucher specimen deposited in museum collections for the lower Tocantins River, including 299 species with voucher specimens for the THR influence area. During our 2005–2007 field campaigns, we recorded 404 species and provided our own digital vouchers for 275 species (68%), 140 of which currently have archived vouchers in either WikiAves (82 species represented by images) or xeno-canto (84 species represented by sound-records). Seventy-five species were also photographed following captures by mist-netting. Only 26 species that we recorded are not currently represented by a museum specimen, image, or sound-record; 23 of which were recorded during our quantitative surveys (point counts, mist-netting, or boat census); and 3 species recorded only by either visual or acoustic observations: *Panyptila cayennensis*, *Threnetes leucurus* and *Micrastur mirandollei*. We decided to retain these species in the final checklist because of their high reliability of occurrence considering our experience and their known geographic and ecological distribution. Another two recorded species are considered as hypothetical due to inadequate level of documentation: *Jabiru mycteria* and *Hydropsalis maculicaudus* (Table S1). We recorded 107 species for the first time for THR avifauna. Most of these species are residents, 47 species are classified as migrants and 23 with unexpected occurrences may be vagrants, occur as low-density migrants or are expanding their ranges in Amazonia following deforestation (Appendix S1).

According to the IUCN Red List (IUCN, 2019) and the Brazilian Threatened Species List (ICMBio, 2018), a total of 44 (9.2%) threatened species were confirmed as part of the THR avifauna, 34 of which still persist in this area. Most of these threatened species are endemic to the Amazonian biogeographic provinces known as the Belém Endemism Area or the Xingu Endemism Area, east and west of the Tocantins River, respectively. *Crax fasciolata pinima* (Belém Curassow) and *Phlegopsis nigromaculata paraensis* (Black-spotted Bare-eye) were not recorded during the 2005–2007 inventory. These are both subspecies from Belém Endemism Area red-listed as Critically Endangered and Vulnerable, respectively. As a replacement for the Belém Curassow, the subspecies *Crax fasciolata fasciolata* (Bare-faced Curassow) was recorded on both banks of the THR influence area, suggesting that some left bank individuals had been translocated to the THR right bank during the rescue operations as the reservoir filled. In a similar way, we obtained three photos from two records of *Ortalis superciliaris* (Buff-browed Chachalaca) at the Tucuruí municipality on the left-bank. It is unclear if these records represent an ecologically viable population from translocated individuals or a natural expansion of the geographic distribution.

**Figure 2:**
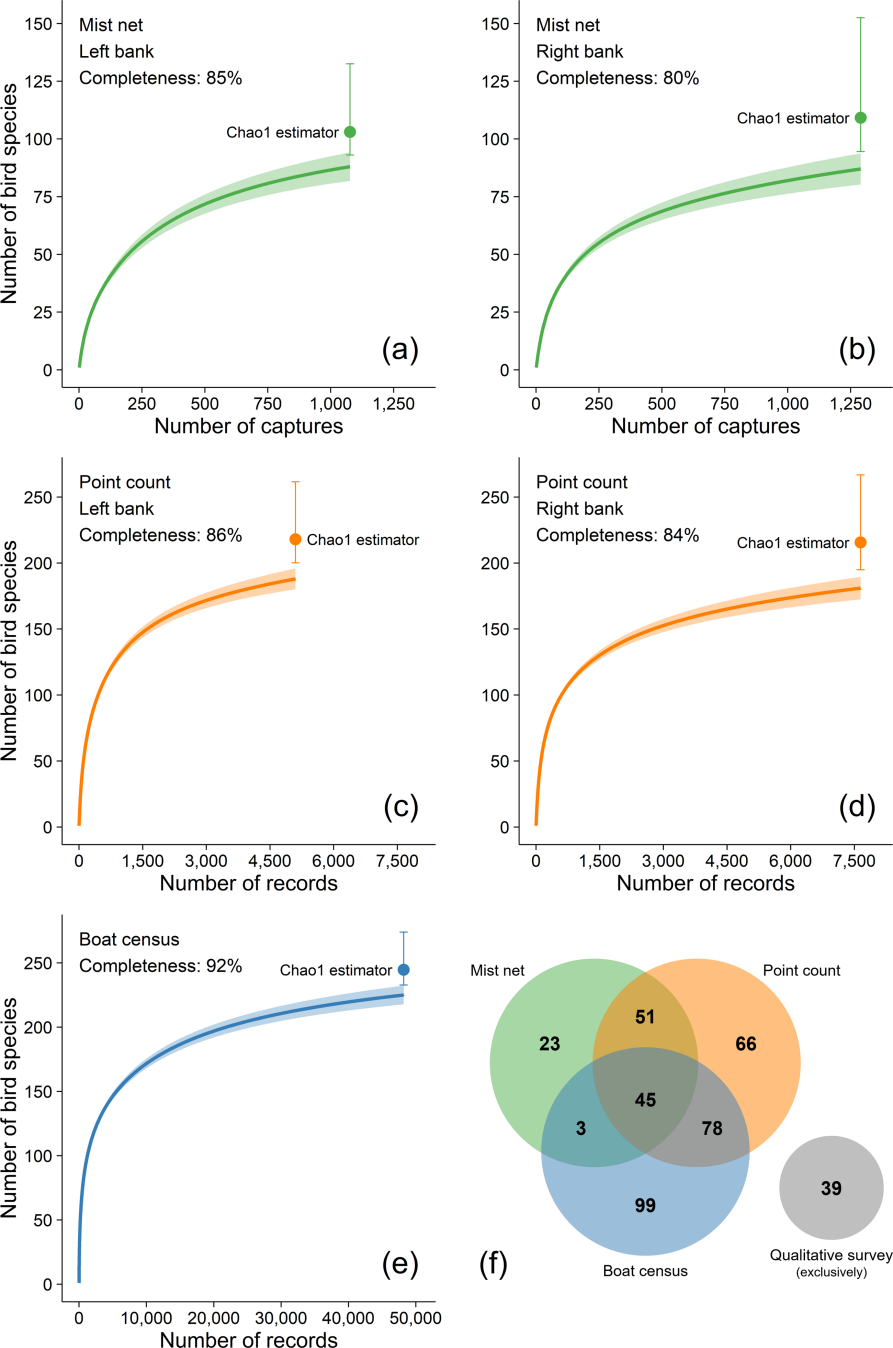
(A-E) Individual-based rarefaction curves with 95% confidence intervals of the number of bird species at the Tucuruí Hydroelectric Reservoir influence area. Sampling completeness per riverbank was quantified as a percentage between the recorded and the estimated number of bird species based on the Chao1 estimator (Chao, 1984).

Our species accumulation curves are near asymptotic for all sampling techniques (Fig. 2). The total number of estimated species produced by the Chao1 estimator indicates a total sampling completeness of 80–85% for mist-netting, 84–86% for point-counts and 92% for boat censuses (Fig. 2). These three quantitative survey techniques have inherent biases toward some species groups and are affected by habitat characteristics, but their complementary use increased the total richness as indicated by the number of species uniquely recorded by each method: 23, 66, and 99 species were exclusively recorded by mist-netting, point-counts, and boat censuses, respectively. Another 39 species were exclusively recorded by our qualitative effort. This effort resulted in a total of 404 species (316 species on the left bank and 307 on the right bank). Furthermore, recent sighting records made by 47 volunteers who recorded a total of 358 species were searched using online citizen science databases and added to our overall effort.

### Possible extinctions on THR influence area

We failed to record 75 species for the THR influence area, 53 of which (11.06% of the THR avifauna) were considered as extinction-prone candidates (Table 1). We excluded two species considered as vagrants (*Pterodroma incerta* and *Stercorarius pomarinus*), one exotic species (*Passer domesticus*) and 12 species with digital vouchers on WikiAves, whose records are the first for the THR avifauna (Appendix S1). We also excluded seven species recorded by volunteers after 1984 with archived vouchers in WikiAves, e-Bird or xeno-canto: *Picumnus aurifrons* (two records in 2017 and one record in 2018); *Pyrrhura anerythra* (one record of one group with three individuals in 2020); *Sakesphorus luctuosus* (three records - 2018, 2019 and 2020); *Myrmoborus leucophrys* (one record in 2018); *Cranioleuca vulpina* (one record in 2019); *Iodopleura isabellae* (one record in 2015); *Pachyramphus rufus* (four records: 2013, 2017, 2018, 2020). These seven species cannot be considered extinct, but the number of sighting records suggest that their populations are declining and may be locally extinct in large portions of the THR influence area.

We were able to estimate extinction probabilities for 20 species (for which the number of historical records was ≥ 5). A total of 15 species were considered to be extirpated based on Solow’s equation and nine species were presumed to be extant (Table 1). *Psophia interjecta*, an endemic of the Xingu Endemism Area that was split from *Psophia viridis* (Ribas et al., 2012), and *Pyrilia vulturina*, a target species in the Brazilian wild bird trade, are listed as Endangered and Vulnerable in the IUCN Red List, respectively (IUCN, 2019). For eight species the extinction dates ranged from 1980 to 2003 and their extinction events most likely directly result from the implementation of the Tucuruí Dam. Nine species are strictly dependent on forest habitat, including three that are restricted to river-edge or other fluvial habitats that were critically impacted by the dam and the rising floodwaters: *Hypocnemoides melanopogon*, *Nasica longirostris* and *Automolus rufipileatus*. Two other species, *Gymnomystax mexicanus* and *Nemosia pileata*, are also found in riverine habitats and, together with *Chlorestes notatus*, *Manacus manacus* and *Chiroxiphia pareola*, are common species in old secondary forests and degraded primary forests.

**Table 1:** Species that were not recorded in the last 36 years after damming of the Tocantins River in 1984 when the Tucuru Hydroelectric Reservoir were created.

**Species**	**Habitat** ^a^	**Microhabitat** ^b^	**Sociality** ^c^	**Diet** ^d^	**Xingu River** ^e^	**Last Record**	**n** ^f^	**tn** ^g^	**T** ^h^	***Solow*** ^i^	***Estimated year***	***Upper 95% bound***
*Zebrilus undulatus*	af	w	s	pi	3/14°	1912	1					
*Ixobrychus exilis*	w	w	s	pi		1984	1					
*Psophia interjecta*	tf, af	t	mf	om		1984	16	51	87	0.000	1987	1993
*Accipiter poliogaster*	tf	c	s	in		1984	1					
*Nyctiprogne leucopyga*	w	ef	s, mf	in	3/9°	1984	2					
*Podager nacunda*	w	w	s, mf	in		1907	1					
*Avocettula recurvirostris*	af	m	s	ne	1/16°	1916	5	8	113	0.000	1916	1921
*Lophornis gouldii*	af	c	s	ne		1912	3					
*Chlorestes notata*	sg, tf, af	u	s	ne		1931	35	71	113	0.000	1980	1985
*Heliomaster longirostris*	tf, af	c	s	ne		1912	2					
*Nonnula ruficapilla*	af	u, m	s	in	2/10°	1984	1					
*Picumnus cirratus*	tf, af, sg	c, ef	s	in		1978	5	71	113	0.158		
*Piculus leucolaemus*	tf, af	m	s, uf	in		1912	1					
*Pyrilia vulturina*	tf, af	c	mf	fr	46/49°	1984	13	72	108	0.008	1989	2003
*Myrmornis torquata*	tf	t	s	in		1984	4					
*Epinecrophylla leucophthalma*	tf	u, m	s, uf	in	11/3°	1984	25	77	113	0.000	1987	1993
*Myrmotherula multostriata*	af	u	s, uf	in	125/4°	1984	7	77	113	0.099		
*Hypocnemoides melanopogon*	af	u	s	in	116/3°	1931	13	23	110	0.000	1935	1939
*Hylophylax punctulatus*	tf, af	u	s	in	79/3°	1984	2					
*Myrmelastes rufifacies*	tf, af	u	s	in	3/2°	1916	2					
*Conopophaga melanogaster*	tf	u, t	s	in		1912	4					
*Hylopezus berlepschi*	sg	t, ef	s	in	34/8°	1984	1					
*Xiphorhynchus obsoletus*	af	u, ef	s, uf	in	22/11°	1985	8	78	113	0.074		
*Campylorhamphus multostriatus*	tf	c, m	s, uf	in	7/10°	1984	6	77	113	0.145		
*Nasica longirostris*	af	c	s	in	9/4°	1931	6	19	108	0.000	1944	1944
*Automolus rufipileatus*	af	u	s	in	12/9°	1931	15	74	113	0.002	1989	2001
*Anabacerthia ruficaudata*	tf, af	u, m, c	s, uf	in	3/5°	1912	1					
*Philydor pyrrhodes*	tf	u	s	in	5/5°	1984	6	72	108	0.135		
*Certhiaxis cinnamomeus*	af, sg	ef	s	in		1984	4					
*Manacus manacus*	sg, tf	u	s, l	fr		1984	24	74	110	0.000	1987	1993
*Chiroxiphia pareola*	tf, sg	u, m	s, l	fr	3/3°	1931	35	21	110	0.000	1932	1933
*Myiobius atricaudus*	af	u	s, uf	in	1/5°	1984	4					
*Pachyramphus viridis*	sg	ef, tf	s	om		1985	1					
*Phoenicircus carnifex*	tf	c, m	s, l	fr		1916	6	9	113	0.000	1917	1922
*Corythopis torquatus*	tf	u, t	s	in		1984	1					
*Elaenia chilensis*	sg	c, ef	s	om		1907	3					
*Elaenia chiriquensis*	sg, w	c	s	om		1916	1					
*Capsiempis flaveola*	sg, w	ef	s	in		1984	3					
*Serpophaga hypoleuca*	sg, w	ef	s	in		1907	1					
*Cnemotriccus fuscatus*	sg, af	u, ef	s	in		1984	1					
*Vireolanius leucotis*	tf	c, m	s, cf	in	34/6°	1912	2					
*Pygochelidon melanoleuca*	w	a, w	mf	in	1208/2°	1984	3					
*Atticora fasciata*	w	a, w	mf	in	273/2°	1984	1					
*Turdus fumigatus*	af	u, m	s	om	17/2°	1984	9	74	110	0.041	1992	2013
*Psarocolius viridis*	f	c	mf	om		1922	3					
*Gymnomystax mexicanus*	w	u	mf	om		1931	7	24	113	0.000	1934	1943
*Tangara gyrola*	tf	c, ef	cf	fr		1907	2					
*Nemosia pileata*	af, sg	m	s, cf	in		1931	12	24	113	0.000	1933	1940
*Chlorophanes spiza*	tf	c	cf	om		1931	3					
*Lanio cristatus*	tf, af	c, m	s, cf	om	4/5°	1984	18	74	110	0.001	1988	1996
*Cyanerpes caeruleus*	tf, af	c	s, cf	om		1916	4					
*Cyanerpes cyaneus*	tf, af	c	s, cf	om	1/38°	1916	4					
*Euphonia minuta*	tf, af	c	cf	om		1016	3					

**Notes.**

a Habitat: tf, terra firme forest; af, alluvial forest; w, open areas near water (beaches and rock outcrops seasonally flooded); sg, secondary growth.

b Microhabitat: w, near water; c, canopy; m, midstory; u, understory; t, terrestrial; ef, forest edge.

c Sociality: s, solitary or pair; cf heterospecific canopy flocks; uf - heterospecific understory flocks; mf, monospecific flocks; l, leks.

d Diet: pi, piscivores; in, insectivores; fr, frugivores; om, omnivorous; ne, nectarivores.

e Number of records in the Belo Monte Hydroelectric Dam Area of Influence before the Xingu River was dammed and number the days until the first record.

f Number of records in the lower Tocantins River.

g The interval between the first and the last record.

h The time interval between the year of the first sighting and the target year (2007). The p values shown are based on the Solows equation (Solow 1993, 2005).

i The *p* values shown are based on the Solows equation (Solow, 1993; Solow, 2005).

Another 67 species recorded for lower Tocantins River, either upstream or downstream of the THR influence area, were missing from our surveys (Table S1). These species were distinguished into five groups: (1) species recorded downstream of the THR; (2) species restricted to natural savannah habitats, with low probability of occurrence in THR; (3) species recorded upstream of the THR in the Marabá municipal county; (4) species recorded both upstream and downstream of the THR; and (5) species recently recorded in any of the municipal counties around the THR influence area. We are reasonably confident that most of these highly forest-dependent species once occurred throughout the THR influence area but have now become locally extinct.

### Extinction risk

**Figure 3:**
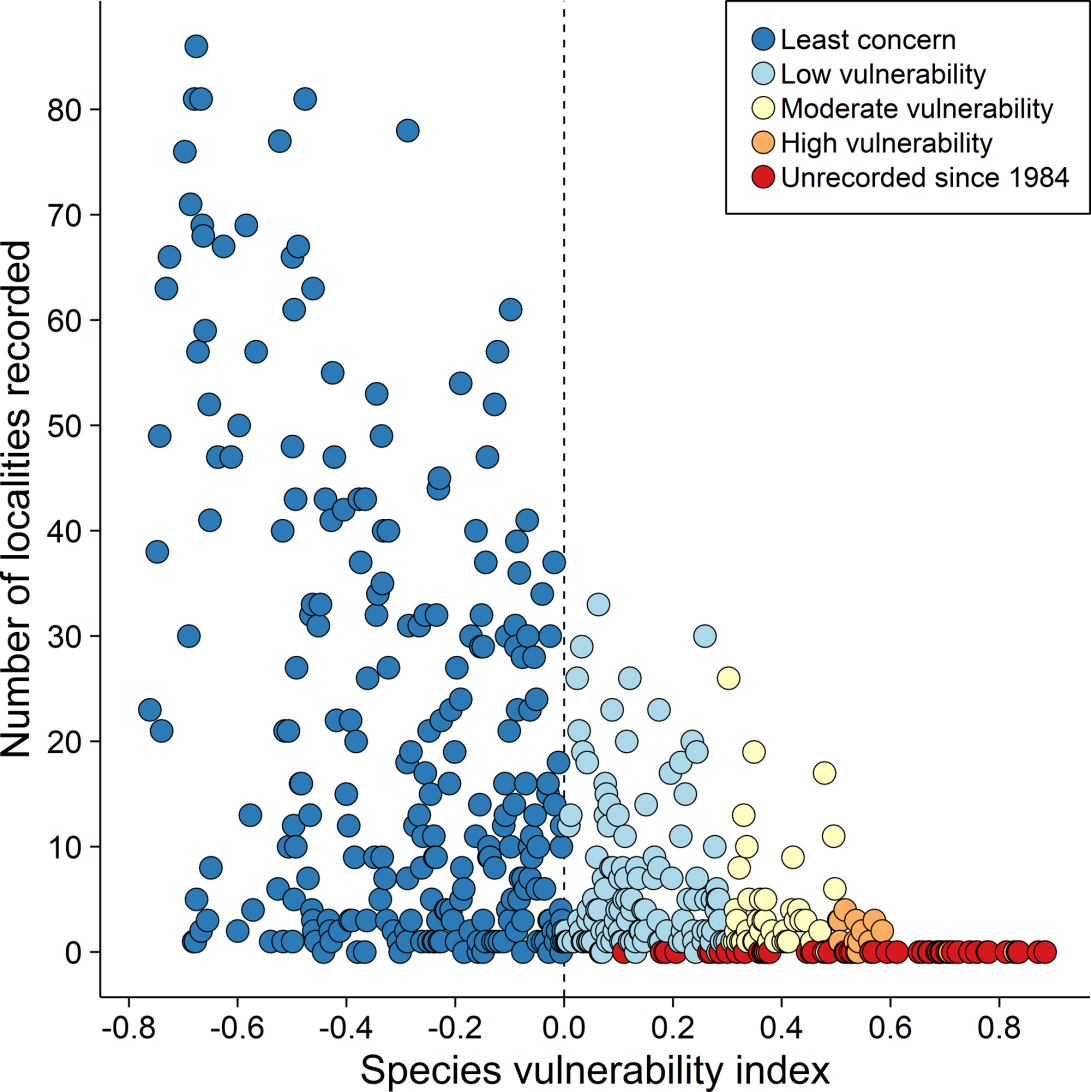
Extinction risk for 476 species of the Tucuruí Hydroelectric Reservoir *vs.* number of sites with confirmed occurrence (132 sites: 39 forest islands and 93 reservoir margins segments). The Vulnerability index (Phi) was based on four criteria related to rarity (habitat breadth, geographic range size, abundance, and relationship between the expected and observed occurrence). The species were grouped into four categories according to Phi intervals: Least concern (dark blue: all species with negative Phi); Low vulnerability (light blue: species with 0.3 >Phi >0.0); Moderate vulnerability (light yellow: species with 0.5 >Phi >0.03); High vulnerability (orange: species with Phi >0.5). All species unrecorded since 1984 were marked with red and they spanned the entire vulnerability gradient.

Our extinction vulnerability assessment indicates that 54% of the 236 species classified as vulnerable through a combination of criteria related to rarity were recorded in fewer than seven of the 139 sites on the THR influence area that we surveyed and 30% were recorded in only one or two (Fig. 3 and Table S2). Considering a set of 133 vulnerable species recorded in 39 forest islands with different areas, we visually identified four broad occurrence categories (Fig. S3). First, species restricted to forest islands larger than 1,800 ha, including terrestrial and understory insectivorous species such as *Microcerculus marginatus*, *Sclerurus rufigularis*, and obligate ant following *Dendrocincla merula* and *Phlegopsis nigromaculata*. This category also included the midstory insectivore *Jacamerops aureus* and the canopy frugivore *Deroptyus acciptrinus*. The second category included species occurring on forest islands larger than 100 ha and also understory and midstory insectivores, such as *Piprites chloris*, *Platyrynchus saturatus*, the small frugivorous *Lepidothryx iris* and *Dixiphia pipra*. The third category included those species whose occurrence included islands smaller than 100 hectares despite a low abundance (*e.g.*, *Conopophaga roberti*, *Phaethornis superciliosus*). The fourth category included highly abundant species occurring across the forest island gradient surveyed (*Lophotriccus galeatus*, *Pteroglossus aracari*, *Thamnophilus stictocephallus*). The first three categories illustrate the potential decline of forest-dependent populations and, consequently, the increase in regional scale extinction risk due to the high degree of deforestation within and around the THR. The species in our extinction-prone candidate list were classified as Vulnerable, but they spanned the entire vulnerability gradient.

## Discussion

### Avifaunal inventory effort and data limitations: general overview

Here, we present a comprehensive landscape-scale avifaunal inventory effort focused on the THR influence area after 21–23 years since the onset of maximum reservoir inundation. Our inventories draw upon different and complementary sampling methods, which tend to survey bird communities in heterogeneous habitat mosaics most efficiently. Our sampling protocol was based on quantitative techniques (mist-netting, point counts, and boat censuses) supplemented by qualitative efforts involving less systematic ad hoc field observations. This effort resulted in a total of 404 species (316 species on the left bank; 307 on the right bank), with over 100 species recorded for the first time for the avifauna of this region. This high number of new records occurred in part because previous studies had focused on more haphazard collections of voucher specimens using mist-nets and firearms. Both methods are highly selective and were used with little regard for establishing a general quantitative baseline from which future compositional changes in the avifauna could be best monitored and assessed. Even studies developed during the construction phase of the hydroelectric dam failed to provide such a quantitative baseline. For example, the book chapter on birds in the Inventory of Fauna Utilization Project (Plano de Inventário do Aproveitamento da Fauna—PIAF, in Portuguese; Eletronorte, 1985a) presented a qualitative checklist, without assessing the complete hydrological cycle and lacking information on sampling effort or number of replicates. The rather expensive and controversial wild vertebrate rescue operation named Operação Curupira (Eletronorte, 1985b), which cost approximately US$30 million to deploy over several years, and contributed little information on the avifauna of this region, since most individuals rescued were relatively large-bodied and often remained unidentified at the species level. Therefore, many relatively common aquatic or canopy-dwelling species had not been recorded in the THR region prior to our study, and we do not have abundance data for most of avian species before the Tocantins River was dammed.

Despite the lack of previous quantitative data, our large sampling effort, with additional recent records from online citizen science databases, indicates that remarkable changes occurred in the THR avifauna after a 36 years of post-inundation history with an increase in extinction risk as consequence of the synergistic effects of flooded, deforestation and forest degradation surrounding the reservoir. Even confirming the large number of bird species persisting in the long term, our forest island occupancy analysis indicates that several forest species classified as vulnerable are locally extinct on forest islands smaller than 1,800 ha or smaller than 100 ha. This scenario can be extended to the entire THR influence area, even though the open-water matrix is more inhospitable than newly created anthropogenic habitats surrounding the reservoir (Mendenhall et al., 2014). Conversely, we recorded open-habitat species that were unexpected in this region, such as *Athene cunicularia*, *Columbina squammata*, *Zenaida auriculata*, *Amazona aestiva*, *Myiothlypis flaveola* and *Gnorimopsar chopi*. Although at low abundances, these open-habitat species have been gradually expanding their geographic ranges following deforestation. This gradual turnover consisting of local extinctions of forest specialists and replacements by open-habitat species has been documented elsewhere along the ‘Arc of Deforestation’ (*e.g.,* Moura et al., 2013).

### Species extinction after 36 years of post-inundation history

In a presence-absence analysis of species extinction of the THR, Henriques & Dantas (2009) suggested that most of the missing species in our sampling effort and part of the species historically recorded only downstream or upstream of the reservoir have in fact been extirpated (71 species with 27 additional extinct species on the right bank alone). They emphasized that 70% of these species are forest specialists, including 30% of riverine habitat specialists that were completely eliminated by the rising floodwaters. However, this may have been overestimated because some of these species were recorded only upstream or downstream of the THR influence area or may not belong to the regional species pool. In fact, although species presence records are assumed to be reliable, the same could not be said for absence records as failing to detect any given species does not necessarily equate to absences (*e.g.*, Gu & Swihart, 2004). We found evidence for the possible extinction of 53 consistently recorded species for the THR influence area, all of which remained undetected since 1984, including *Cnemotriccus fuscatus*, erroneously listed as still occurring in THR by Bueno et al. (2018). We can also confirm the vulnerability of forest specialist species, particularly insectivores and fluvial habitat specialists, including river species associated with sand banks, river islands, and rocky outcrops (Table 1), which are usually highly vulnerable habitats to hydroelectric development (Lees et al., 2016). Specialized forest insectivores frequently show high sensitivity to habitat fragmentation (Stouffer & Bierregaard, 1995; Sekercioglu et al., 2002; Powell, Cordeiro & Stratford, 2015), even though other life history traits, such as large body size, specialized feeding habits, low dispersal capacity and narrow geographic distribution, are good predictors of bird sensitivity to extinction (*e.g* Bregman, Sekercioglu & Tobias, 2014).

Our extinction probability estimates for 20 species (for which the number of historical records was ≥ 5) indicate that (i) 15 species (75%) have gone extinct in the THR influence area, (ii) part of these extinctions is related to the impacts of land-use change prior to damming, but (iii) the rate of species loss increased from 0.25 species/year from 1916 to 1944 to 0.34 species/year from 1980 to 2003. To put this in perspective, in a long-term (>160 years) analysis of bird community erosion of the region around the eastern Amazonian city of Belém, the rate of species loss was estimated at 0.28 species/year (Moura et al., 2014). The extinction rate may be even higher in the THR influence area since we are confident that other species were also last detected in 1984, and several extinction-prone species are restricted to riverine habitats that were permanently flooded by the rising floodwaters in the aftermath of river damming. Most of these habitat specialist species are in fact visually or acoustically conspicuous, and very unlikely to be overlooked over three years of intensive sampling. For example, we recorded several of these species during less than 60 days of fieldwork as part of the Xingu River surveys using the same sampling methods (Table 1). However, our data also indicate that *Myrmotherula multostriata*, *Xiphorhynchus obsoletus* and *Campylorhamphus multostriatus* may still be extant, even though their habitat had been permanently flooded. In a study on methods for inferring species extinction based on sight records, Boakes, Rout & Collen (2015) show that Solow’s equation provides low predictive power if extinction time is near the end of the observation period, which suggests that these species may now be extinct according to Solow’s equation (Table 1).

Considering species for which we were unable to calculate extinctions probabilities (those with fewer than 5 historical records), we are confident that *Hylophylax punctulatus* and *Hylopezus berlepschi*, two alluvial forest species, *Vireolanius leucotis*, one canopy *terra-firme* forest, *Pygochelidon melanoleuca* and *Atticora fasciata,* two gregarious habitat-specialist species of rocky outcrop habitats, are also locally extinct given our field experience with these species at other Amazonian sites that indicate they are common and conspicuous. The alluvial forest and the rocky outcrop habitats were extirpated by permanent flooding and high deforestation rates of upland forest. A recent multitemporal analysis of deforestation in response to the construction of the Tucuruí Dam indicates the highest deforestation rate occurred in the first period of the analysis (1984–1988), due to the areas submerged by the reservoir and due to the anthropogenic disturbances, such as timber extraction, road construction, and the conversion of large tracts of forests into croplands (Velastegui-Montoya, De Lima & Adami, 2020). The other 25 species with fewer than five records include one globally threatened species and one near-threatened (IUCN, 2019), respectively: *Lophornis gouldii* and *Zebrilus undulatus*. These and several other extinction-prone species in our hypothetical list are conspicuous and very unlikely to go undetected over three years of intensive sampling. These missing species indicate that Solow’s equation as used here likely underestimates the full impact of the Tucuruí Hydroelectric Dam on the avifauna.

Our analysis also revealed the impacts of land-use change in the bird community of THR in the first half of the 20th century, because seven species became presumably extinct between 1916 and 1944. These species were extinct during the course of the construction of the Tocantins Railway, used to transport Brazil nuts and natural rubber. Between the inauguration of the first stretch of the railway in 1908 and the last, 36 years were required to build 117 km, which connected Alcobaça (present-day Tucuruí) to Jatobal, an occupation process that led to the near extinction of three indigenous groups of the lower Tocantins basin (Laraia & Da Matta, 1978). Although these findings are less severe than the impacts of land-use change on the bird community of the San Antonio cloud forest over 100 years (Kattan, Alvarez-Lopez & Giraldo, 1994; Palacio, Kattan & Pimm, 2020), it stresses our scant knowledge about the historical impacts of the modern colonization of Amazonia on biodiversity.

### Implications for the long-term impact assessments of hydropower infrastructure

Our results show that (1) the lack of a pre-damming systematic inventory likely masks some of the temporal changes in extinction patterns; (2) it is critical to initiate conservation measures *before* the main impact, when vulnerable species can be more easily surveyed; and (3) further studies and the creation of protected areas beyond the immediate area impacted by hydropower infrastructure are critically required to protect habitats and species at the landscape scale.

Our results are consistent with the notion that avian assemblages in human-modified Amazonian forests likely pay a heavy extinction debt over long timescales. Much like the Belém case study (Moura et al., 2014), the conservation value of remaining forest patches at Tucuruí decays over time because disturbance-adapted species are recruited into novel assemblages at the expense of the gradual loss of forest-dependent species (a typical case of the ‘winner-loser’ paradigm: Filgueiras et al., 2021). We expect that insular forest patches created by the reservoir typically harbored depauperate avian assemblages comprised primarily of naturally abundant and habitat generalist species (see also Bueno et al., 2018). Biodiversity loss in Amazonia is largely irreversible and a consequence of deforestation, and the THR influence area is no exception because of the elevated number of threatened bird species (Bird et al., 2011). However, other threats have also been detected over the 36 years since the damming of the Tocantins River, such as illegal logging, forest wildfires, overhunting and the spread of domestic mesopredators (*e.g.,* cats and dogs) (Barlow et al., 2006; Whiteman et al., 2007; Benítez-López et al., 2017). The synergistic effects of these threats coupled with forest habitat loss clearly aggravates avian population declines and local extinctions in the Tucuruí region.

The high levels of avian gamma-diversity hosted by the THR places it among the most diverse across the Amazon basin. Contributing to this high diversity is the fact that the THR influence area includes portions of both the Xingu and Belém lowland areas of endemism, with several parapatric species replacing one another on opposite riverbanks. This diversity can be higher if we consider infra-specific taxa and recent molecular studies, which confirm deep molecular divergence between opposite riverbanks (*e.g.*, Ribas et al., 2012 for *Psophia* complex; Batista et al., 2013 for *Dendrocolaptes certhia* complex; Maldonado-Coelho et al., 2013 for *Pyriglena leuconota* complex). In addition to this high diversity, we recorded 44 threatened or near-threatened species (IUCN, 2019; ICMBio, 2018), 34 of which are still extant in the study region. These species have been further threatened by increasing habitat loss since the dam was built (see Fig. 1), highlighting the relevance of this region for biodiversity conservation.

## Conclusions

Our avifaunal inventory in the THR influence area serves as a baseline from which changes in species composition—driven by the short to long term impacts of this major hydroelectric dam—can be better monitored and assessed over a large landscape. Our species extinction estimates highlight the fact that non-random habitat loss and the degree of habitat specialization of species under consideration are key determinants of species extinctions. They also highlight that avian species extinction will continue to occur in the THR influence area as a result of direct impact of seasonally flooded alluvial forest loss and the continued degradation of the remaining *terra firme* forests (Chen et al., 2015). Although the specific mechanisms driving extinctions vary across species, the essential point is that riparian habitats were permanently lost to inundation by the hydroelectric dam in the short term and that highly fragmented landscapes do not bode well in the long term for any extant forest avifauna (Lees & Peres, 2006). Changes in the THR avifauna will continue to occur, including the continued decline of populations of forest species that were probably relatively common before 1984 and increase of habitat generalists and open-countryside species. Therefore, our study identifies some conservation management implications that should be considered both before and after the construction of new hydroelectric dams: (1) large dams result in local vertebrate extinctions. Thus, it is critical to begin deploying conservation measures *before* the onset of the expected impacts of major hydropower infrastructure, when the most vulnerable species are easier to detect; this may include protection of vulnerable habitats and species both within the area immediately impacted by the dam reservoir and surrounding landscapes; and (2) the conservation measures must been supported by quantitative faunal inventories initiated at the planning stages of new dams and then followed by long-term monitoring plans.

The Tucuruí region may represent much of the future of lowland Amazonia under a business-as-usual scenario. The time lag between the construction of the Tucuruí Dam and the proper implementation of protected areas as a mitigation measure calls for an urgent and comprehensive forest restoration plan. It is critically necessary to increase the connectivity between small forest fragments in this highly fragmented landscape, which should result in larger population sizes for several species, thereby reducing the chances of future extinctions. Despite the fact that hydropower is the prevailing energy source in the Brazilian Amazonia, this is the first study that investigates the long-term impacts of major hydroelectric dams on Amazonian birds. This would not have been possible without a time series including pre-inundation databases, even if we account for potential historical collecting biases. Our results also suggest that previous bird studies on the impact of archipelagic landscapes created by Amazonian dams (Balbina Hydroelectric Reservoir: Aurélio-Silva et al., 2016; THR: Bueno et al., 2018) have underestimated the long-term ecological impacts of hydroelectric dams on local to regional-scale extinction rates. These studies lack a proper baseline and consider only the post-flooding history, when several species are already extirpated at landscape scales. Understanding the long-term impacts of major dams and other infrastructure on tropical forest biodiversity will therefore remain a critical priority in lowland Amazonia, particularly considering ambitious investments in hydropower blueprints from central government for future development.

## Supplemental Information

10.7717/peerj.11979/supp-1 Supplemental Information 1 History of ornithological exploration of lower Tocantins River Supplemental Materials and Figures

10.7717/peerj.11979/supp-2 Supplemental Information 2 Appendix 1 and Supplemental Tables

## Additional Information and Declarations

### Field Study Permissions

The following information was supplied relating to field study approvals (i.e., approving body and any reference numbers): Secretaria Executiva de Ciencia, Tecnologia e Meio Ambiente do Estado do Para - SECTAM provided full approval for this research (license number 013/2005) and Museu Paraense Emilio Goeldi (Projeto Fauna de Tucurui - Convenio Museu Goeldi/Eletronorte).

### Data Availability

The following information was supplied regarding data availability: The raw data and materials are available in the Supplemental Files.
