# Supplementary material for: Avian extinctions induced by the oldest Amazonian hydropower mega dam: evidence from museum collections and sighting data spanning 172 years
Source: PeerJ. 2021 Aug 18;9:e11979. doi: 10.7717/peerj.11979 (PMC8380028; doi:10.7717/peerj.11979)
Supplement: Supplemental Information 1 — Supplemental Materials and Figures [file peerj-09-11979-s001.docx]

**SUPPLEMENTAL INFORMATION**

**Avian extinctions induced by the oldest Amazonian hydroelectric mega dam: evidence from museum collections and sighting data spanning 172 years**

Luiza Magalli Pinto Henriques, Sidnei M. Dantas, Lucyana Barros Santos, Anderson S. Bueno, Carlos A. Peres

*PeerJ*

**History of ornithological exploration of lower Tocantins River**

The existing literature consists of citations found in catalogues of the Brazilian and Amazonian avifauna and a few checklists based on historical avian collections. Alfred Russel Wallace (1853), who pioneered avian studies along the Tocantins River, collected 26 avian specimens “between the mouth of the river and the first falls” and the collection, deposited at the British Museum of Natural History, was examined by Sclater & Salvin (1867). Emilie Snethlage working at the Museu Paraense Emílio Goeldi (MPEG) began intensive sampling expeditions in the early 20th century that collected birds on both the left and right banks of the lower Tocantins River. In 1907, she collected birds in the localities of Alcobaça (now Tucuruí) and Arumateua, on the left bank, some 25 km south of Tucuruí. In 1910, she worked in the right-bank municipality of Baião, including some localities near this town (Igarapé Pirinum, Igarapé Boca do Manapiri and Igarapé Pai Lourenço). In 1911, she worked with F. Lima in the left-bank municipality of Cametá. In 1912, F. Lima collected birds at the left-bank localities of Arumateua and Alcobaça, and the right-bank locality of Mazagão. These efforts resulted in a collection of 1020 specimens, including those used to describe three new species to science, *Campylorhamphus multostriatus*, *Hemitriccus minor* and *Hemitriccus griseipectus*, and a complete summary of all ornithological material collected during this period (Snethlage, 1914). The specimens Snethlage reports are largely deposited at MPEG, but part of the material was exchanged with the American Museum of Natural History and other ornithological collections in the USA and Europe. Finally, F. Lima collected birds in 1916 at the left-bank municipality of Cametá, including a sampling effort in the savanna vegetation (localities Fazenda Vaicajó and Campo de Pucurijó).

Other professional collectors also worked along the Tocantins River. For example, A. M. Olalla collected for the American Museum of Natural History in 1931 at Baião and Mocajuba, on the right bank, and Ilha do Taiúna. In 1933, he collected birds for the Harvard Museum of Comparative Zoology in Cametá, on the left bank. His collections added over 900 specimens, including the type specimen of *Oxyruncus cristatus* *tocantinsi*, and records of this species along the lower Tocantins River were later interpreted as altitudinal migration (Silva, 1993). An anonymous 1920 collection from Marabá and Sítio Floresta, housed at the Harvard Museum of Comparative Zoology, added 58 specimens. In 1978, during the construction phase of the Tucuruí Dam, M. Moreira collected birds at Sítio Calandrini, Tucuruí; about 170 specimens were deposited at the MPEG.

From February to June 1984, during the pre-flooding stage of the reservoir, an intensive sampling effort was conducted by a MPEG team with the participation of Maria Luiza Videira, David Oren, Fátima Lima and Manuel Santa Brigida. They collected birds at several localities on the left (Cocal, Jacundá, Vale do Caraipé, Vila Temporária I, Vila Permanente, Igarapé Saúde, Timbozal, Praia Alta, Remansão da Beira and Jatobal) and right banks (Canoal and Jacundazinho), and on river islands of the Tocantins River (Ilha Tocantins and Ilha Tangerina). This effort resulted in a collection of 630 specimens housed at the MPEG Ornithological Collection (Eletronorte 1985a). Silva et al. (1990) described this checklist, including the body mass of each specimen. Also, as part of the effort funded by Eletronorte, a pre-damming wildlife rescue operation called *Operação Curupira* was conducted between 1984–1985, when over 4000 birds were rescued. Unfortunately, most of these birds were not identified at the species level, but included 1012 cracids, 305 tinamids and 215 raptors, including a Harpy Eagle (*Harpia harpyja*). Among those species identified at the species level, there were 1185 individuals of *Opisthocomus hoazin*, 18 of *Psophia interjecta* and 13 of *Psophia obscura* (Eletronorte 1985b).

From 2005 to 2007, within the cooperation agreement between Eletronorte, Agencia Nacional de Energia Eletrica (ANEEL) and MPEG, Luiza Magalli Henriques, Sidnei Dantas, Lucyana Barros and Cesar Cestari developed studies for the establishing of a bird monitoring program at APA Lago de Tucurui (Cestari & Dantas, 2008; Henriques & Dantas, 2009; Bueno et al., 2018).

Downstream Tucuruí Reservoir, Alexander Lees and Nárgila Moura developed studies on non-forest bird species using edaphic savanna vegetation (*campina*) along the lower Tocantins River (Lees et al., 2014).

In the last years, with the popularization of three initiatives that increase citizen science in Amazonia (WikiAves, xeno-canto and e-Bird), we assessed data from 47 birdwatchers in the various municipalities surrounding the Tucuruí Hydroeletric Reservoir. They are making new records and records of some species whose populations have been dramatically reduced around the Tucuruí Hydroeletric Reservoir. We hope that our compiled Checklist of Tucuruí Hydroeletric Reservoir Avifauna and discoveries related to avian extinction risk give base for the intersection of citizen science and the long term biodiversity monitoring of this region.

**References**

Henriques LMP, Dantas SM. 2009. Composição e Extinção Local de Espécies na Comunidade de Aves da APA Tucuruí, Entorno do Reservatório da UHE Tucuruí, Amazônia Oriental. In: *Anais do V Congresso de Inovação Tecnológica em Energia Elétrica*, Belém, Para, MA-4. Available at http://www.aneel.gov.br/cds/-/asset_publisher/z7Us6TO39fU3/content/v-congresso-de-inovacao-tecnologica-em-energia-eletrica-v-citenel/656835?inheritRedirect=false (accessed November 2018).

Cestari C, Danta, SM. 2008. Registros de psitacídeos ameaçados de extinção no lago da usina hidrelétrica de Tucuruí, Estado do Pará. *Atualidades Ornitológicas*. 145: 8-9.

Sclater PL, Salvin O. 1867. List of birds collected by Mr. Wallace on the lower Amazonas and Rio Negro. *Proceedings of the Zoological Society of London*, 1867: 556–596.

Wallace AR. 1953. *A narrative of travels on the Amazon and Rio Negro, with account of the native tribes and observation on the climate, geology, and natural history of the Amazon valley*. London. Reeve & Co. 541p.

Snethlage E. 1914 - Catálogo das Aves Amazônicas. *Boletim do Museu Paraense Emílio Goeldi de História Natural e Etnografia*, 8: 1–530.

Silva JMC da, Lima MFC, Marceliano MLV. 1990. Pesos de Aves de duas localidades na Amazônia Oriental. *Ararajuba*, 1: 99–104.

Silva JMC da. 1993. The sharpbill in the Serra dos Carajás, Pará, Brazil, with comments on altitudinal migration in the Amazon Region. *Journal of Field Ornithology*, 64: 310–315.

Eletronorte. 1985a. *Plano de Enchimento do Reservatório: Fauna - Projetos Faunísticos em Tucuruí*. Brasília. vol. 1.

Eletronorte. 1985b. *Plano de Enchimento do Reservatório: Fauna – Análise da Operação Curupira.* Brasília. vol. 2.

Bueno AS, Dantas SM, Henriques LMP, Peres CA. 2018. Ecological traits modulate bird species responses to forest fragmentation in an Amazonian anthropogenic archipelago. *Diversity and Distributions*, 24: 387–402.

Lees AC, Moura NG, Almeida AS, Vieira IC. 2014. Noteworthy ornithological records from the threatened campinas of the lower rio Tocantins, east Amazonian Brazil. *Bulletin of the British Ornithologists’ Club*, 134(4), pp.247-258.

**List of Excel Spreadsheets**

**Appendix 1.** Checklist of Tucuruí Hydroelectric Reservoir Avifauna

Legend for Appendix 1 and List of Photographers and collaborators of WikiAves, xeno-canto and e-Bird.

**S1.** Hypothetical species list of Tucuruí Hydroelectric Reservoir.

**S2.** Extinction Risk Ranking.

**S3.** Sampling Points (2005-2007).

**S4.** Census Stretches (2005-2007)

**S5.** Historical Collection Points (1907-1984).

**S6.** Land Cover of Tucuruí Hydroelectric Reservoir

**
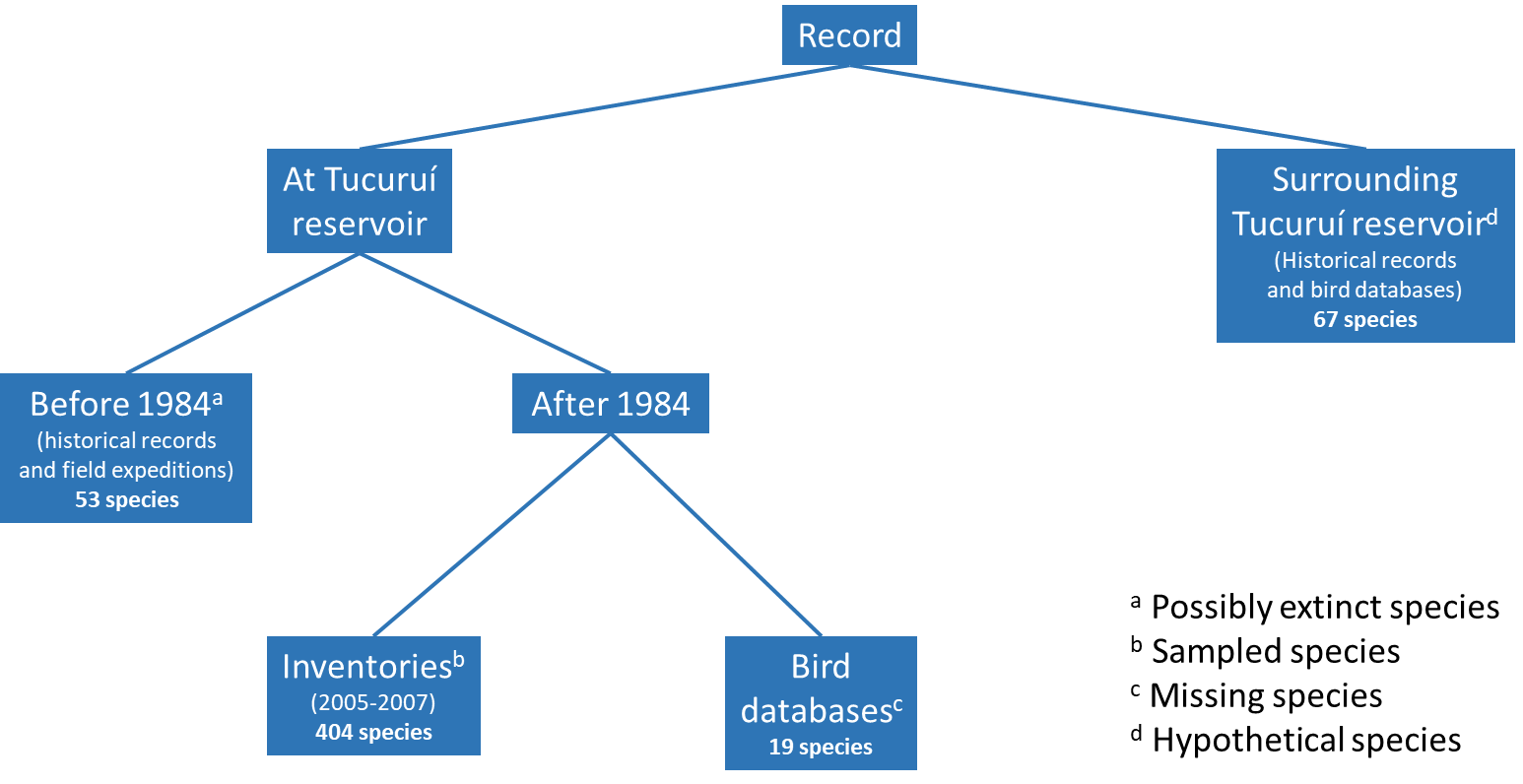
**

**Figure S1.**  Comprehensive mind map for Tucuruí Reservoir Avifauna


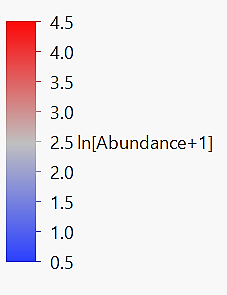


**(A)**

**(B)**

**Figure S2.** Site-by-species incidence matrix for 133 bird species surveyed across 39 islands at the Tucuruí Hydroelectric Reservoir landscape. Islands are ordered from the largest to the smallest.
